# Anesthetic management of a 12-year-old child with Kagami-Ogata syndrome for pectus excavatum: a case report

**DOI:** 10.1186/s40981-020-00397-6

**Published:** 2020-11-15

**Authors:** Hisako Nishimoto, Masahiro Yagihara, Aki Uemura, Yoshiki Nakajima

**Affiliations:** 1 Department of Anesthesiology and Intensive Care, Hamamatsu University Hospital, 1-20-1 Handayama, Higashi-Ku, Hamamatsu City, Shizuoka 431-3192 Japan; 2 Department of Anesthesiology, Anshin Hospital, 1-4-12 Minatojimaminamimachi, Chuo-Ku, Kobe City, Hyogo 650-0047 Japan

**Keywords:** Kagami-Ogata syndrome, Pectus excavatum repair, Anesthetic management, Postoperative respiratory failure

## Abstract

**Background:**

Kagami-Ogata syndrome (KOS) is due to abnormal gene expression in the 14q32.2 imprinted region. Laryngomalacia and bell-shaped thorax of children with KOS can affect airway management of general anesthesia.

**Case presentation:**

A 12-year-old girl with KOS had a mechanical ventilation history and underwent pectus excavatum repair for cosmetic reasons. Although she had undergone invasive thoracic surgery under general and epidural anesthesia, her respiratory rate and tidal volume were stable with adequate pain control mainly through epidural analgesia at the end of the surgery. We examined her larynx by a bronchoscope. Then, we successfully extubated her after confirming the normal movement of her larynx.

**Conclusions:**

When patients with KOS undergo pectus excavatum repair, anesthesiologists should prevent postoperative respiratory failure by providing adequate postoperative analgesia. Evaluation of airway patency and respiratory pattern before extubation is critical.

## Background

Kagami-Ogata syndrome (KOS) is caused by abnormal gene expression at the 14q32.2 imprinted region [1]. Its manifestations include a small, bell-shaped thorax with “coat-hanger”-shaped ribs, peculiar craniofacial and cervical features, mental retardation, and laryngomalacia [1, 2].

Yamagata K et al. reported the successful anesthetic management of 2-year-old child with KOS complicated with marked tracheal deviation and small bell-shaped thorax who underwent orchiopexy for bilateral cryptorchidism [3]. In the case report, they paid attention to preoperative evaluation of the respiratory tract and used laryngeal mask airway as the airway device under consideration of preoperative respiratory condition, tracheal deviation, and surgical procedure [3].

The following report describes an older child with KOS who underwent general anesthesia for pectus excavatum repair. This work has been reported in line with the CARE guidelines [4].

## Case presentation

A 12-year-old girl with KOS (weight 30 kg, height 140 cm) was scheduled for a pectus excavatum repair for cosmetic reasons. She has a family history of KOS. She was born at 30 weeks gestation with a birth weight of 1546 g, and had been kept in a neonatal intensive care unit (NICU) for 4 months. During her NICU stay, she presented with respiratory failure from her thoracic configuration and required mechanical ventilation. She underwent a tracheostomy at 3 months and a gastrostomy for feeding at 3 years. At 6 years of age, the tracheostomy was closed and the gastrostomy tube was removed. These procedures were all uneventful.

**Fig. 1:**
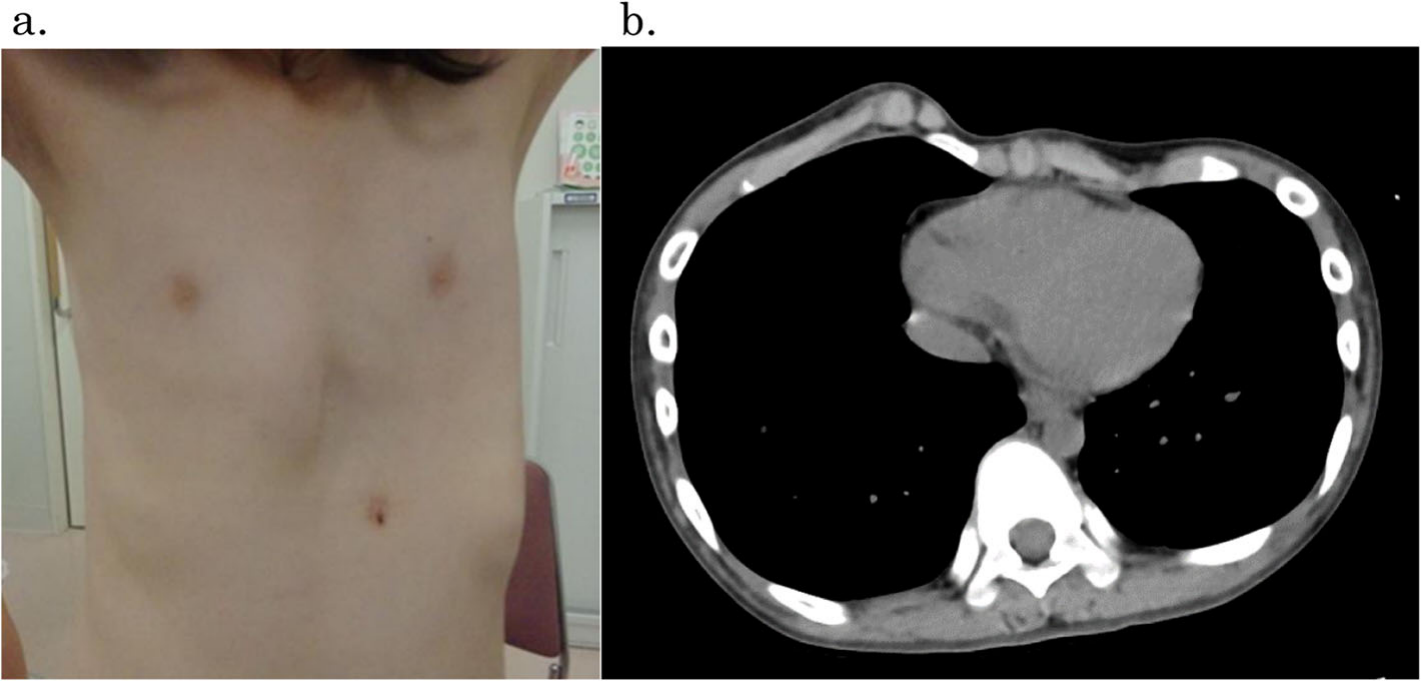
**a** Appearance. Asymmetric pectus excavatum can be observed. **b** Preoperative computed tomographic scan of the chest. Computed tomographic scan shows pectus excavatum deformity

She had no physical symptoms and complications associated with the prior tracheostomy. Several craniofaciocervical features were noted such as frontal bossing, depressed nasal bridge, full cheeks, anteverted nares, protruding philtrum, micrognathia. and short webbed neck. Although she had mental retardation, she was able to follow simple instructions given by us and make simple conversation. She did not have symptoms such as dyspnea, fatigue, or precordial pain after exercise despite remarkable chest wall asymmetry (Fig. 1). Sternocostal elevation was scheduled for pectus excavatum.

Anesthesia was planned as a combination of general and epidural anesthesia. With no premedication, general anesthesia was induced with 8 % sevoflurane with 6 L/min O_2_ via a facemask. Snoring occurred during a slow inhalation induction. The endotracheal tube (φ5.5 cm) was easily inserted. Under general anesthesia, an epidural catheter was inserted via the T7–T8 intervertebral space without any difficulty. General anesthesia was maintained with 2% sevoflurane, remifentanil, and rocuronium. Local anesthetics (0.25% levobupivacaine and 0.2% ropivacaine) were administered from the epidural catheter, a total of 29 ml of levobupivacaine was administered intermittently during operation. Epidural patient-controlled analgesia was initiated with 5 ml/h of 0.2% ropivacaine, with a 3 ml bolus dose and 30-min lockout time. The volume of 0.2% ropivacaine injected during operation was 18 ml.

Her sternocostal elevation was initiated with a median vertical incision. Pediatric surgeons resected parts of her costal cartilages (3rd to 8th on the right; 2nd to 8th on the left), adjusted them to appropriate lengths, and reattached them to the sternum. Then, the lower part of the sternum was resected. The surgery was uneventful and lasted 4 h 40 min. Blood loss was 202 ml.

We measured her spontaneous breathing pattern, while keeping a sufficient depth of anesthesia (2% sevoflurane and 0.7 μg/kg/h dexmedetomidine). At the end of surgery, we used 60 mg sugammadex to reverse the neuromuscular block. She presented with respiratory rate of 20 breaths/min, tidal volume of 200 ml, and end tidal CO_2_ of 43 mmHg. Her respiratory status was stable. Before extubation, we performed a bronchoscope to confirm edema and/or stenosis in the pharyngolaryngeal region. Bronchoscopic examination did not show pharyngolaryngeal edema and stenosis.

Tracheal extubation was performed 30 min after administering 0.7 μg/kg/h dexmedetomidine. Bronchoscopy was performed soon after extubation to evaluate vocal cords and detect laryngomalacia. Dexmedetomidine was continued for sedation and analgesia during bronchoscopic examination. The movement of the vocal cords and larynx was normal; a floppy epiglottis was not observed. She was then transferred to the ward. The anesthesia time was 6 h 25 min. Her post-operative recovery was uneventful. She was discharged on the 9th day after her surgery.

## Discussion

We were concerned that our patient’s underlying disease coupled with the invasive surgery might cause postoperative respiratory failure. Therefore, our anesthetic management focused first on adequate postoperative pain relief. We performed epidural analgesia for preventing possible respiratory problems resulting from mental retardation and laryngomalacia which are often seen in KOS [2, 5], although epidural anesthesia was not required for analgesia after sternocostal elevation according to previous reports [6, 7].

Next, our anesthetic management focused on safe extubation. Specifically, we performed a bronchoscope before and soon after extubation to evaluate airway patency. We measured her respiratory pattern before extubation to evaluate respiratory parameters and respiratory depression.

We also attempted to prevent postoperative agitation after awakening from general anesthesia. Kain ZN et al. reported that emergence delirium was likely to be caused by several variables, including a child’s underlying temperament, preoperative anxiety, use of some anesthetics and other drugs, and other conditions such as pain [8]. Because the patient was developmentally disabled, we were anxious about postoperative agitation. Dexmedetomidine is an α_2_-adrenoceptor agonist with sedative, anxiolytic, sympatholytic, and analgesic-sparing effects, and minimal depression of respiratory function [9]. Ibacache ME et al. showed that dexmedetomidine administered after inducing anesthesia reduced post-sevoflurane agitation in children, with no adverse effects [10]. In this case, dexmedetomidine prevented emergence agitation when administered prior to recovery from general anesthesia.

Our patient had several craniofaciocervical features. In particular, micrognathia and short webbed neck are associated with airway management. Micrognathia is concerned with anterior mandibular space. Any condition that makes the space small relative to the size of the tongue will make tracheal intubation more difficult [11]. Short webbed neck is concerned with flexion and extension of the neck. Flexion and extension of the neck is necessary to align the axes of the trachea and oral cavity to provide a line of sight for intubation [11]. Although our patient had micrognathia and short webbed neck, these craniofaciocervical features did not make tracheal intubation difficult to perform. It is unclear how craniofaciocervical features change with growth.

In July 2020, we searched two major databases (e.g., PubMed, Google Scholar) to identify relevant articles, “Anesthetic management of Kagami-Ogata syndrome.” The initial search terms were Kagami-Ogata syndrome and general anesthesia. No date restrictions and research design filters were imposed on the searches. We excluded articles published in abstract form, in a language other than English. One article was found on the initial literature search. The article was “Anesthetic management of a child with Kagami-Ogata syndrome complicated with marked tracheal deviation: a case report” on PubMed and Google Scholar. The article reported on anesthetic management of 2-year-old child with KOS complicated with marked tracheal deviation and small bell-shaped thorax [3]. In patients with KOS, various degrees of thoracic abnormality change with increasing age and small bell-shaped thorax tends to ameliorate with age [2]. In the course of treatment for KOS, mechanical ventilation was performed in most patients and tracheostomy was carried out in about one-thirds of patients [2]. Unlike the earlier reported case of 2-year-old child with KOS, it is likely that anesthetic management of older children with KOS is not required to consider the effect of small bell-shaped thorax. However, it is necessary to consider the clinical severity and degree of pectus excavatum and the effect of the treatments the patient have received, i.e., mechanical ventilation and tracheostomy.

KOS is a rare disease, with fewer than 100 patients in Japan [5]. Only one study has been reported on anesthetic management of patients with KOS. In addition, the study reported on anesthetic management of 2-year-old child with KOS. However, considering the survival rate of children with KOS (73.5%) [2], the number of surgical procedures for older children with KOS such as our case is likely to increase.

## Conclusion

When patients with KOS undergo pectus excavatum repair, anesthetic management should focus on providing adequate postoperative analgesia, preventing postoperative respiratory failure, and avoiding emergence agitation. Evaluating the respiratory pattern and airway patency before and soon after extubation is critical.

## Data Availability

Not applicable

## Abbreviations

KOS: Kagami-Ogata syndrome
NICU: Neonatal intensive care unit
